# Effects of Different Feeding Modes on Growth Performance, Blood Biochemistry, and Metabolism of Yushu Yaks During the Cold Season

**DOI:** 10.3390/ani16071110

**Published:** 2026-04-03

**Authors:** Chengeng Liang, Hai Hu, Guowen Wang, Shangrong Xu, Shi Shu, Rong Huang, Changqi Fu, Wei Peng

**Affiliations:** Academy of Animal and Veterinary Sciences, Qinghai University, Xining 810016, China

**Keywords:** blood biochemistry and metabolism, feeding modes, growth performance, Yushu yaks

## Abstract

Yushu yaks face nutritional deficiency and poor production performance in the cold season on the Qinghai–Tibet Plateau, yet their metabolic responses to different feeding modes remain unclear. This study investigated the effects of traditional grazing, grazing with concentrate supplementation, and TMR stall-feeding on 90 four-year-old Yushu yaks (30 per group) during a 180-day cold-season trial, by determining body weight changes, serum biochemical parameters and plasma metabolic profiles. Stall-fed yaks had the highest average daily gain (259.05 ± 61.56 g/d), while grazing yaks showed negative gain (−279.50 ± 44.45 g/d) and supplementary-fed yaks had intermediate performance; grazing yaks had stronger antioxidant enzyme activity, and supplemented/stall-fed yaks had higher serum mineral and albumin levels. Metabolomic analysis identified 2024 plasma metabolites, with grazing yaks enriched in plateau adaptation and antioxidant pathways, stall-fed yaks in energy metabolism pathways, and supplementary-fed yaks in both growth and antioxidant pathways. Our findings confirm that gradient nutritional interventions regulate the balance between yak growth and plateau adaptability, with concentrate supplementation as the optimal cold-season feeding mode for local herders and stall-feeding suitable for intensive breeding to maximize growth efficiency.

## Simple Summary

Yushu yaks living above 4000 m on the Qinghai–Tibet Plateau face insufficient nutrition in natural pastures. Their production performance and health conditions are poor during the cold season, yet their adaptive metabolic responses to different feeding patterns remain unclear. To address this, we assigned 90 four-year-old yaks to three groups (grazing, grazing + supplementary feeding, stall-feeding) for a 180-day cold-season trial, to explore the effects of feeding modes on their growth, blood biochemistry and metabolism. Results showed stall-fed yaks had the highest daily weight gain, while grazing yaks lost weight; grazing yaks had stronger antioxidant enzyme activity, and supplemented/stall-fed yaks had higher mineral and albumin levels. We first identified 2024 metabolites in Yushu yaks, finding that nutritional intervention exerted a gradient effect on their metabolism, with supplementary feeding showing intermediate metabolic characteristics. Grazing yaks were enriched in plateau adaptation-related pathways, stall-fed yaks in energy metabolism, and supplemented yaks in both growth and antioxidant pathways. This study clarifies how supplementary feeding balances nutrition and plateau adaptability, providing practical feeding guidance for local herdsmen and supporting the sustainable development of the alpine yak industry.

## 1. Introduction

The yak (*Bos grunniens*) is a ruminant unique to the Qinghai–Tibet Plateau. Through long-term evolution, it has developed excellent adaptability to extreme environmental conditions such as high altitude, low temperatures, and hypoxia [1,2]. Studies have found that yaks have positive selection in genes related to energy metabolism, oxygen transport, and thermoregulation, which is the core molecular basis for their adaptation to high-altitude environments with nutrient deficiency and hypoxia. A unique population of lung endothelial cells has been identified in yaks, which enhances the contractility of the lungs and the efficiency of oxygen utilization, further elucidating the physiological adaptation mechanism of yaks to hypoxia at the cellular level [3]. As a rare livestock breed in the Yushu area (altitude > 4000 m), the Yushu yak not only provides essential products for the local herdsmen’s livelihood but also plays a crucial role in maintaining the ecological balance of alpine grasslands [4]. However, the breeding of Yushu yaks is highly dependent on the traditional grazing mode in natural pastures, and it faces severe challenges in the cold season. The withering of forages in winter leads to a scarcity of available forage and a decline in nutritional quality (comprehensive net energy is only 3.54 MJ/kg, and crude protein is 6.12%), resulting in physiological problems such as weight loss in yaks, which further restricts the sustainable development of the yak industry [5].

With the rapid development of modern animal husbandry, supplementary feeding and pen-feeding modes have been adopted in the breeding of Yushu yaks. Research has shown that optimizing feeding methods can improve the growth performance of yaks, enhance nutrient utilization efficiency, and alleviate cold stress [6]. Zhu et al. [7] found that increasing the protein level in the diet can significantly improve the average daily gain of growing yaks in the cold season. An appropriate concentrate-to-roughage ratio can promote the growth of pen-fed yaks, improve rumen fermentation, and regulate blood metabolic levels, thus alleviating the nutritional stress of yaks. Ma et al. [8] discovered that a high-energy diet can increase the total volatile fatty acid concentration in the rumen of yaks in the cold season by 18.6% and significantly up-regulate the expression of genes related to nutrient absorption in the rumen epithelium. Wang et al. [9] systematically revealed the adaptation mechanism of yaks to high-altitude environmental stress through multi-omics analysis, finding that their overall metabolic level changes significantly and the gut microbiota plays a key role in the nutrient assimilation of yaks under high-altitude conditions. Xue et al. [10] demonstrated through lipid metabolomic analysis that supplementary feeding can improve the energy supply of grazing yaks by regulating lipid metabolism. Some research has found [11] that supplementary feeding with concentrates in the cold season can significantly increase the relative abundances of Firmicutes and Prevotella in the yak rumen, enhance the metabolic pathways of amino acid biosynthesis and carbohydrate degradation, and further clarify the regulatory effect of supplementary feeding on yak rumen fermentation.

Therefore, this study takes Yushu yaks as the research object, systematically compares the impacts of different feeding methods on their growth performance and blood biochemical indicators, and for the first time clarifies the core metabolic pathways and the optimal feeding method for Yushu yaks in winter under extreme high-altitude conditions. Most existing metabolomic studies are mainly limited to the identification of differential metabolites and fail to integrate plateau-adaptation-related pathways to explain the balance mechanism between yak growth and environmental adaptation under different feeding methods [12]. This research gap has led to a lack of breed-specific theoretical guidance for the winter feeding of Yushu yaks, which are adapted to extreme high-altitude environments above 4000 m [13]. Clarifying the metabolic response characteristics of Yushu yaks to different intensities of nutritional intervention is of great significance for optimizing local yak feeding strategies and promoting the sustainable development of the alpine yak industry [14].

We hypothesized that the three winter feeding modes (grazing, grazing with supplementary feeding, and pen-feeding) would cause significant differential changes in the growth performance, blood biochemical parameters, and metabolic phenotypes of Yushu yaks. Nutritional intervention would have a distinct gradient effect on the metabolic regulation of Yushu yaks adapted to extreme high-altitude environments. The supplementary-feeding mode would balance the improvement of their nutritional status and the maintenance of their plateau-adaptive antioxidant capacity by regulating the core metabolic pathways of Yushu yaks.

This study aims to systematically compare the effects of three typical winter feeding modes (grazing, grazing with supplementary feeding, and pen-feeding) on the growth performance, blood biochemical indicators, and metabolic characteristics of Yushu yaks adapted to extreme high-altitude environments above 4000 m. Meanwhile, it aims to identify the core differential metabolic pathways of Yushu yaks in response to different feeding methods and nutritional intervention intensities, and further reveal the molecular mechanism by which supplementary feeding balances the growth performance and plateau-antioxidant capacity of Yushu yaks. Ultimately, this study is expected to fill the research gap in the metabolomics of high-altitude endemic yak breeds and provide a breed-specific theoretical basis and practical technical references for the scientific feeding and management of Yushu yaks in alpine pastoral areas during winter.

## 2. Materials and Methods

### 2.1. Experimental Design

This experiment was conducted from December 2024 to May 2025 in Qumalai County, Yushu Tibetan Autonomous Prefecture, Qinghai Province (altitude: approximately 4200 m). The experimental period coincided with the typical cold season in this alpine region, with the average daily temperature ranging from −15 °C to 5 °C. Precipitation mostly occurred in the form of snow, and the atmospheric oxygen content was approximately 60% of that at plain levels. A total of 90 healthy four-year-old Yushu yaks with similar body weights (278.27 ± 38.36 kg) and good body conditions were selected. The male-to-female ratio was balanced (half males and half females), and all female yaks were non-pregnant to eliminate the confounding interference of pregnancy on experimental indicators and ensure that the feeding mode was the only variable among groups. The experimental period was from December 2024 to May 2025. During this stage, all female yaks were in anestrus, and the male yaks used in the experiment were intact, non-castrated bulls. The selected yaks were randomly divided into three groups, with 30 yaks in each group (15 males and 15 females), namely the grazing group, the grazing plus supplementary-feeding group (referred to as the supplementary-feeding group for short), and the stall-feeding group. The trial included a 14-day pre-test adaptation period during which all groups were managed uniformly, followed by a 180-day formal experimental period.

### 2.2. Rearing Management

The grazing group was raised under the local traditional grazing mode, with free access to natural forages and drinking water on the native pasture at an altitude of approximately 4200 m (92°56′ E~97°35′ E, 33°36′ N~35°40′ N) from 08:30 h to 18:30 h daily. The feeding sheds for the supplementary feeding and stall-feeding groups were located in the same area, which ensured the same altitude across all three groups and thus eliminated environmental interference. The supplementary-feeding group received supplementary group feeding of 1.0 kg of concentrate per head per day after daily grazing, with free access to drinking water ad libitum. The stall-feeding group was reared in stalls for the entire trial period and fed a total mixed ration (TMR) with a concentrate-to-forage ratio of 3:7 at 08:30 h and 18:30 h daily, with ad libitum access to feed and drinking water at each feeding time.

### 2.3. Diet Formulation and Nutritional Levels of Forage

A complete pelleted concentrate was formulated based on the nutrient requirements of beef cattle specified in the Chinese Feeding Standard for Beef Cattle, adjusted to meet the maintenance and productive requirements of female yaks during the cold season. The concentrate was composed of corn, wheat bran, soybean meal, rapeseed meal, sodium bicarbonate, sodium chloride, a commercial vitamin–mineral premix (BH40-QH, Unipromex, Beijing, China), and a probiotic supplement (Yishengji Powder, Sichuan Hengtong Animal Pharmaceutical Co., Ltd., Neijiang, China). The probiotic supplement contained *Bacillus subtilis* (2 × 1011 CFU/kg), *Bacillus licheniformis* (2 × 1011 CFU/kg), and *Saccharomyces cerevisiae* (2 × 1010 CFU/kg). The complete feed was manufactured by Huanghe Chuxing Animal Husbandry Development Co., Ltd., Xining, China. The composition of dietary formulations is presented in Table 1, and the nutritional levels of diets and natural forage are presented in Table 2.

### 2.4. Body Weight Measurement and Sample Collection

The experimental procedures were carried out in accordance with the regulations of the Laboratory Animal Management Committee of the Academy of Animal Husbandry and Veterinary Sciences, Qinghai University (2024-QHMKY-018), and all methods were implemented in line with the rules and guidelines established by this committee. Yaks in the three groups were weighed following an overnight fast at the beginning (Day 1) and the end (Day 180) of the experiment. The average daily gain (ADG) was calculated based on the initial and final body weights. After the experiment, six yaks (3 males and 3 females) were randomly selected from each group. Before the morning feeding, 10 mL of whole blood was collected from the jugular vein of each fasting yak and separately transferred into test tubes with and without anticoagulant for subsequent examination. The blood samples were allowed to stand for 30 min and then centrifuged at 4000 rpm for 10 min. Serum and plasma were separated and collected for the determination of biochemical parameters and blood metabolomics analysis [15]. All samples were immediately snap-frozen in liquid nitrogen and subsequently stored in a −80 °C ultra-low temperature freezer until analysis.

### 2.5. Determination of Biochemical Parameters

The following biochemical parameters were determined using a Hitachi 7600-020 automatic biochemical analyzer (Hitachi High-Technologies Corporation, Tokyo, Japan) and enzyme-linked immunosorbent assay (ELISA) kits (purchased from Wuhan Elabscience Biotechnology Co., Ltd., Wuhan, China) [16]: alanine aminotransferase (ALT), aspartate aminotransferase (AST), creatinine (CREA), lactate dehydrogenase (LDH), total protein (TP), albumin (ALB), globulin (GLOB), glucose (GLU), triglyceride (TG), high-density lipoprotein cholesterol (HDL), low-density lipoprotein cholesterol (LDL), alkaline phosphatase (ALP), *γ*-glutamyl transferase (*γ*-GT), sodium (Na), potassium (K), chloride (Cl), phosphorus (P), iron (Fe), magnesium (Mg), urea (UREA), uric acid (UA), total cholesterol (T-CHO), malondialdehyde (MDA), total superoxide dismutase (T-SOD), catalase (CAT), glutathione peroxidase (GSH-PX), total antioxidant capacity (T-AOC), and blood urea nitrogen (BUN).

### 2.6. Plasma Metabolomics Analysis

The metabolite extraction method was as follows: 100 μL aliquots of plasma were taken, and 400 μL of extraction solution (80% aqueous methanol, Sinopharm Chemical Reagent Co., Ltd., Shanghai, China) were added. The mixtures were vortexed thoroughly, followed by incubation on ice for 5 min, and then centrifuged at 15,000× *g* and 4 °C for 15 min using a low-temperature centrifuge (D3024R, Scilogex, Rocky Hill, CT, USA). An aliquot of the supernatant was diluted with mass spectrometry-grade water (Merck KGaA, Darmstadt, Germany) to a methanol concentration of 53%, and centrifuged again at 15,000× *g* and 4 °C for 15 min. The resulting supernatant was collected for liquid chromatography–tandem mass spectrometry LC-MS/MS analysis (Novogene Bioinformatics Technology Co., Ltd., Beijing, China; Q Exactive™ HF/Q Exactive™ HF-X, Thermo Fisher Scientific, Waltham, MA, USA) [17]. Pooled quality control (QC) samples were prepared by mixing equal volumes of all plasma samples, and 53% aqueous methanol was used as the blank sample [18]. These QC and blank samples were injected periodically to monitor the analytical stability of the LC-MS/MS system. LC-MS/MS analysis was performed using a Thermo Vanquish UHPLC system (Thermo Fisher Scientific, Waltham, MA, USA) equipped with a Hypersil Gold column (Thermo Fisher Scientific, Waltham, MA, USA; 100 × 2.1 mm, 1.9 μm) [19]. Mobile phase A consisted of 0.1% aqueous formic acid (Sigma-Aldrich, St. Louis, MO, USA), and mobile phase B was pure methanol. The gradient elution program was set as follows: 0 min (98% A), 1.5 min (98% A), 3 min (15% A), 10 min (0% A), 10.1 min (98% A), with a total run time of 12 min. Different gradient profiles were applied for the positive and negative ion modes, respectively. The mass spectrometry conditions were optimized using an electrospray ionization (ESI) source under the following parameters: spray voltage of 3.5 kV, sheath gas flow rate of 35 psi, auxiliary gas flow rate of 10 L·min^−1^, ion transfer tube temperature of 320 °C, and auxiliary gas heater temperature of 350 °C. Data acquisition was conducted in the data-dependent acquisition (DDA) mode, covering a mass range of m/z 100–1500. A mixture of multiple stable isotope-labeled internal standards (including L-2-chlorophenylalanine, D-sorbitol, and 13C6-sucrose, Sigma-Aldrich, St. Louis, MO, USA) was added to the plasma samples prior to metabolite extraction for relative quantification to correct for systematic errors during sample pretreatment and LC-MS/MS analysis.

### 2.7. Data Processing and Statistical Analysis

This study adopted a one-factor completely randomized experimental design, with the feeding mode (grazing, grazing + supplementary feeding, stall-feeding) as the only fixed factor, and the individual yak as the experimental unit. All statistical analyses were conducted with a significance level of *p* < 0.05.

#### 2.7.1. Preprocessing of Metabolomics Data

Raw mass spectrometry data were converted to the mzXML format using ProteoWizard (v3.0.2319, ProteoWizard Team, CA, USA). Chromatographic peak extraction, quantification, and peak alignment were subsequently performed using the XCMS toolbox (v3.8.1, Scripps Research Institute, La Jolla, CA, USA) [20]. Metabolite identification was achieved by matching the accurate mass-to-charge ratio (m/z mass tolerance ≤ 10 ppm) and tandem mass spectrometry (MS/MS) spectra against high-quality reference databases (KEGG, HMDB, LIPID MAPS). Background ions were removed by subtracting signals detected in blank samples. Raw quantitative data were normalized using the following formula to obtain the relative peak area: relative peak area = (raw quantitative value of the metabolite in the sample) ÷ (sum of quantitative values of all metabolites in QC1 sample) × sum of quantitative values of metabolites in the sample. Compounds with a coefficient of variation (CV) of relative peak area > 30% in quality control (QC) samples were excluded. The final dataset containing identified metabolites and their relative quantifications was generated for subsequent analysis.

#### 2.7.2. Statistical Analysis of Bioinformatics

Identified metabolites were annotated using the KEGG, HMDB, and LIPID MAPS databases. Multivariate statistical analyses, including principal component analysis (PCA) and partial least squares discriminant analysis (PLS-DA), were conducted using the metaX metabolomics software package (v2.2.0, Capital Normal University, Beijing, China), and variable importance in projection (VIP) values were calculated [21]. Student’s *t*-test was applied to calculate the *p* value and fold change (FC) between groups. Differential metabolites were screened according to the following criteria: VIP > 1, *p* < 0.05, and FC > 1.2 or FC < 0.833. Visualization analyses were performed in R software (v4.4.1, R Core Team, Vienna, Austria) using specialized packages: the ggplot2 (v3.5.0, Hadley Wickham, Auckland, New Zealand) package for volcano plots, the pheatmap (v1.0.12, Raivo Kolde, Tallinn, Estonia) package for clustering heatmaps, and the corrplot (v0.92, Taiyun Wei, Shanghai, China) package for correlation diagrams. Pathway enrichment analysis was performed based on the KEGG database. A metabolic pathway was defined as significantly enriched when the conditions of x/*n* > y/*N* and pathway *p* value < 0.05 were satisfied (x: number of differential metabolites in the pathway; *n*: total number of metabolites in the pathway annotated in the study; y: total number of differential metabolites in the study; *N*: total number of metabolites annotated in the study).

#### 2.7.3. ANOVA for Blood Biochemical and Weight Gain Data

Statistical analysis of blood biochemical parameters and yak weight gain data was performed using IBM SPSS Statistics 26.0 (IBM Corp., Armonk, NY, USA). One-way analysis of variance (ANOVA) was conducted, and the statistical model was as follows: Yij = μ + αi + εij where Yij = the observed value of the j-th individual in the i-th feeding mode group; μ = the overall population mean of the measured index; αi = the fixed effect of the i-th feeding mode (i = 1, 2, 3, representing grazing, grazing + supplementary feeding, and stall-feeding, respectively); and εij = the random error of the j-th individual in the i-th group, following a normal distribution of N(0, σ^2^).

Multiple comparisons were conducted using Duncan’s multiple range test after verifying the homogeneity of variance and normality of the data. All results were expressed as the mean ± standard deviation (SD). A *p* value of <0.05 was considered statistically significant.

## 3. Results

### 3.1. The Effects of Different Feeding Patterns on Weight Gain of Yaks

As shown in Table 3 and Table 4, the yaks in the stall-fed group achieved the highest weight gain at the end of the experiment, followed by those in the supplementary-fed group; both groups had significantly higher weight gain than the grazing group (*p* < 0.05). Specifically, the average daily gain (ADG) of the stall-fed and supplementary-fed groups showed an increasing trend throughout the experiment, whereas the grazing group exhibited a decreasing trend in ADG over the same period.

### 3.2. Blood Biochemical Parameters

**Figure 1:**
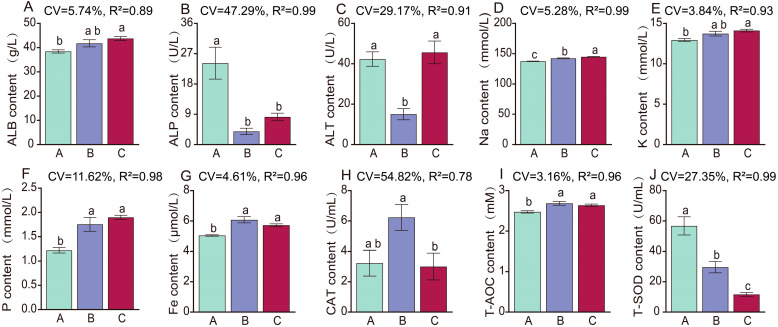
Comparison of significantly different biochemical parameters in yaks (*n* = 6) from the grazing, supplementary-fed, and stall-fed groups. (**A**) Serum alkaline phosphatase (ALP) activity; (**B**) Serum total superoxide dismutase (T-SOD) activity; (**C**) Serum catalase (CAT) activity; (**D**) Serum total antioxidant capacity (T-AOC); (**E**) Serum albumin (ALB) level; (**F**) Serum sodium (Na) concentration; (**G**) Serum phosphorus (P) concentration; (**H**) Serum iron (Fe) content; (**I**) Serum potassium (K) level; (**J**) Serum alanine aminotransferase (ALT) activity. The coefficient of variation (CV) and coefficient of determination (R^2^) of each index are shown in each subplot. Different lowercase letters above the bars indicate significant differences between groups (*p* < 0.05).

Blood biochemical analysis revealed (Supplementary Table S1, Figure 1A–J) that the activities of total superoxide dismutase (T-SOD) and alkaline phosphatase (ALP) in the grazing group were significantly higher than those in the supplementary-fed and stall-fed groups (*p* < 0.05), and T-SOD activity exhibited a gradual decreasing trend in the order of grazing group > supplementary-fed group > stall-fed group. The total antioxidant capacity (T-AOC) of the supplementary-fed group was significantly higher than that of the grazing group, and catalase (CAT) activity was also significantly higher than that of the stall-fed group (*p* < 0.05). The albumin (ALB) level in the stall-fed group was significantly higher than that in the grazing group (*p* < 0.05); the alanine aminotransferase (ALT) activity in the grazing group was significantly higher than that in the supplementary-fed group (*p* < 0.05). Serum sodium (Na) and phosphorus (P) levels in both the stall-fed and supplementary-fed groups were significantly higher than those in the grazing group (*p* < 0.05), and Na concentration showed a gradual increasing trend in the order of grazing group < supplementary-fed group < stall-fed group. The iron (Fe) content in the supplementary-fed group was significantly higher than that in the grazing group (*p* < 0.05); the potassium (K) level in the stall-fed group was also significantly higher than that in the grazing group (*p* < 0.05).

### 3.3. Blood Metabolism

#### 3.3.1. Partial Least Squares Discriminant Analysis (PLS-DA)

**Figure 2:**
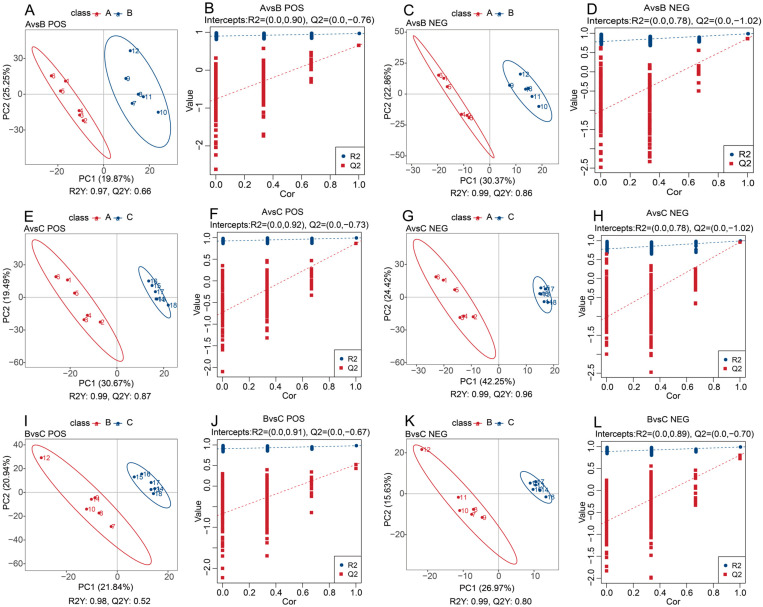
Plasma metabolomic partial least squares discriminant analysis (PLS-DA) score plots and permutation tests of yaks (*n* = 6) from the grazing, supplementary-fed, and stall-fed groups. (**A**) PLS-DA score plot of positive ion mode for grazing vs. supplementary-fed groups; (**B**) Permutation test plot of positive ion mode for grazing vs. supplementary-fed groups; (**C**) PLS-DA score plot of negative ion mode for grazing vs. supplementary-fed groups; (**D**) Permutation test plot of negative ion mode for grazing vs. supplementary-fed groups; (**E**) PLS-DA score plot of positive ion mode for grazing vs. stall-fed groups; (**F**) Permutation test plot of positive ion mode for grazing vs. stall-fed groups; (**G**) PLS-DA score plot of negative ion mode for grazing vs. stall-fed groups; (**H**) Permutation test plot of negative ion mode for grazing vs. stall-fed groups; (**I**) PLS-DA score plot of positive ion mode for supplementary-fed vs. stall-fed groups; (**J**) Permutation test plot of positive ion mode for supplementary-fed vs. stall-fed groups; (**K**) PLS-DA score plot of negative ion mode for supplementary-fed vs. stall-fed groups; (**L**) Permutation test plot of negative ion mode for supplementary-fed vs. stall-fed groups. A, grazing group; B, supplementary-fed group; C, stall-fed group. R^2^Y represents the model explanation rate, Q^2^Y represents the model predictive ability.

Based on PLS-DA analysis, all values of R^2^Y were greater than Q^2^Y, indicating that the constructed model possessed good stability and predictive ability. Moreover, the model could effectively distinguish between Groups A, B, and C in both positive and negative ion modes (Figure 2A–L).

#### 3.3.2. Identification and Classification of Differential Metabolites

**Figure 3:**
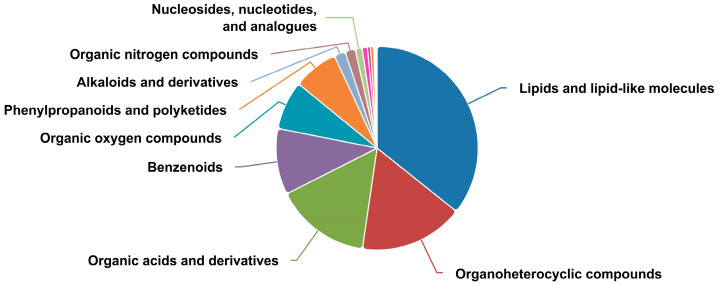
Pie chart for the classification of differential metabolites in the plasma metabolome of yaks (*n* = 6) from the grazing, supplementary-fed, and stall-fed groups.

Based on LC-MS-based metabolomics analysis, a total of 2024 metabolites were identified in positive and negative ion modes, including one thousand two hundred ninety-nine in positive ion mode and seven hundred twenty-five in negative ion mode. According to chemical classification, lipids and lipid-like molecules, organic heterocyclic compounds, and organic acids and their derivatives were the most abundant metabolite classes (Figure 3).

#### 3.3.3. Screening, Identification and Analysis of Differential Metabolites

**Figure 4:**
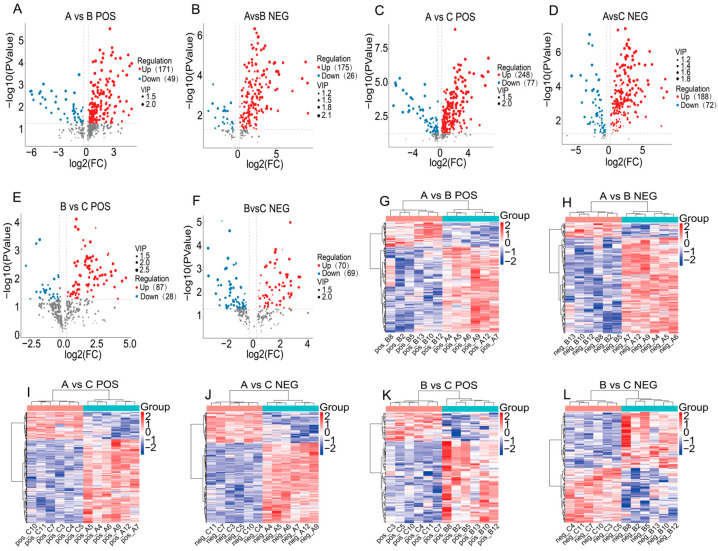
Volcano plots and clustered heatmaps of plasma differential metabolites in yaks (*n* = 6) from the grazing, supplementary-fed, and stall-fed groups. (**A**) Volcano plot of positive ion mode for grazing vs. supplementary-fed groups; (**B**) Volcano plot of negative ion mode for grazing vs. supplementary-fed groups; (**C**) Volcano plot of positive ion mode for grazing vs. stall-fed groups; (**D**) Volcano plot of negative ion mode for grazing vs. stall-fed groups; (**E**) Volcano plot of positive ion mode for supplementary-fed vs. stall-fed groups; (**F**) Volcano plot of negative ion mode for supplementary-fed vs. stall-fed groups; (**G**) Clustered heatmap of positive ion mode for grazing vs. supplementary-fed groups; (**H**) Clustered heatmap of negative ion mode for grazing vs. supplementary-fed groups; (**I**) Clustered heatmap of positive ion mode for grazing vs. stall-fed groups; (**J**) Clustered heatmap of negative ion mode for grazing vs. stall-fed groups; (**K**) Clustered heatmap of positive ion mode for supplementary-fed vs. stall-fed groups; (**L**) Clustered heatmap of negative ion mode for supplementary-fed vs. stall-fed groups. Red dots represent upregulated metabolites, blue dots represent downregulated metabolites (VIP > 1, *p* < 0.05, FC > 1.2 or FC < 0.833); heatmap color intensity indicates the relative abundance of metabolites (red = high abundance, blue = low abundance).Group A is the grazing group, Group B is the supplementary-fed group, and Group C is the stall-fed group.

Differential metabolites among the grazing, supplementary-fed, and stall-fed groups were systematically identified in both positive and negative ion modes. Partial differential metabolites are listed in Supplementary Tables S2 and S3. The most significant metabolic differences were observed between the grazing group and the stall-fed group: 325 differential metabolites were screened in positive ion mode, including 248 upregulated and 77 downregulated ones (Figure 4C,I), and 260 differential metabolites were identified in negative ion mode, with 188 upregulated and 72 downregulated (Figure 4D,J). This was followed by the grazing group versus the supplementary-fed group, where 220 differential metabolites were found in positive ion mode (171 upregulated, 49 downregulated; Figure 4A,G) and 201 in negative ion mode (175 upregulated, 26 downregulated; Figure 4B,H). Minimal metabolic differences were detected between the supplementary-fed group and the stall-fed group, with 115 differential metabolites in positive ion mode (87 upregulated, 28 downregulated; Figure 4E,K) and 139 in negative ion mode (70 upregulated, 69 downregulated; Figure 4F,L). This demonstrates a gradient effect of nutritional interventions on the metabolic phenotypes of yaks under the three feeding patterns.

#### 3.3.4. KEGG Metabolic Pathway Analysis

**Figure 5:**
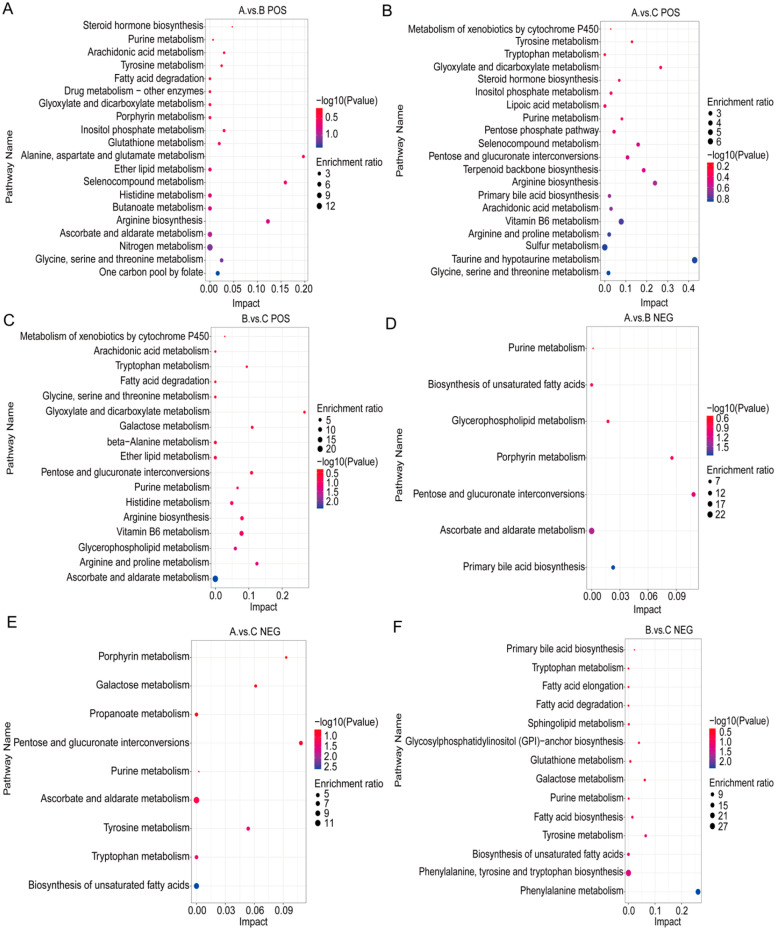
KEGG enrichment bubble plots of the plasma metabolome in yaks (*n* = 6) from the grazing, supplementary-fed, and stall-fed groups. (**A**) KEGG enrichment analysis of differential metabolites in positive ion mode between the grazing group and the supplementary-fed group; (**B**) KEGG enrichment analysis of differential metabolites in positive ion mode between the grazing group and the stall-fed group; (**C**) KEGG enrichment analysis of differential metabolites in positive ion mode between the supplementary-fed group and the stall-fed group; (**D**) KEGG enrichment analysis of differential metabolites in negative ion mode between the grazing group and the supplementary-fed group; (**E**) KEGG enrichment analysis of differential metabolites in negative ion mode between the grazing group and the stall-fed group; (**F**) KEGG enrichment analysis of differential metabolites in negative ion mode between the supplementary-fed group and the stall-fed group. Bubble size represents the number of differential metabolites enriched in the pathway, bubble color depth represents the significance of enrichment (−log_10_(*p*-value)), and the horizontal axis represents the enrichment factor. The pathways are classified into six functional categories: high-altitude adaptation and antioxidant, energy metabolism, amino acid and nitrogen metabolism, lipid metabolism, xenobiotic metabolism and detoxification, and vitamin and secondary metabolism. Different lowercase letters indicate significant differences in pathway enrichment among groups (*p* < 0.05). Group A is the grazing group, Group B is the supplementary-fed group, and Group C is the stall-fed group.

Based on the KEGG enrichment results (Figure 5), the significantly different metabolic pathways among the groups were functionally classified into six categories. The representative pathways and distribution characteristics of each category are as follows: high-altitude adaptation and antioxidant-related pathways were mainly enriched in the grazing group, supporting yaks’ adaptation to high-altitude and cold environments by maintaining cell membrane fluidity and providing antioxidant coenzymes (e.g., ether lipid metabolism, pentose phosphate pathway); energy metabolism-related pathways were predominantly concentrated in the stall-fed and supplementary-fed groups, promoting energy storage and the efficient conversion of nutrients into energy to match the demand for high-energy diets (e.g., fatty acid biosynthesis, galactose metabolism); amino acid and nitrogen metabolism-related pathways exhibited functional differentiation: amino acid catabolism was active in the grazing group, while anabolic pathways were the main enrichment in the supplementary-fed and stall-fed groups (e.g., glycine, serine and threonine metabolism, arginine biosynthesis); lipid metabolism-related pathways were involved in all groups: the grazing group focused on adaptive lipid remodeling, whereas the stall-fed group was dominated by pathways associated with fatty acid deposition (e.g., glycerophospholipid metabolism, biosynthesis of unsaturated fatty acids); xenobiotic metabolism and detoxification-related pathways were mainly enriched in the stall-fed group, reflecting its metabolic adaptation to xenobiotics such as feed additives (e.g., cytochrome P450 metabolism of xenobiotics); and vitamin and secondary metabolism-related pathways showed cross-enrichment among the three groups, correlating with demands for natural forage intake and nutrient conversion, respectively, which reflects the adaptive regulation of secondary metabolism under different feeding patterns (e.g., ascorbate and aldarate metabolism, vitamin B6 metabolism).

## 4. Discussion

### 4.1. Effects of Different Feeding Patterns on the Weight Gain of Yushu Yaks

Yushu yaks inhabit regions at an altitude above 4000 m. During the cold season, they are confronted with the dual environmental stresses of severe cold and low oxygen, with an energy demand far higher than that of ordinary yaks or low-altitude ruminants [22,23]. This is the main reason why the yaks in the grazing group of this study experienced an 18% weight loss. This weight-loss percentage is lower than the over 25% weight loss of grazing yaks in the cold season reported in previous studies [24,25], indicating the inherent adaptability of Yushu yaks to extreme environments and adding new basic data to the research on the growth characteristics of this breed. The low-temperature environment during the cold-season trial increased the maintenance nutritional requirements of yaks, resulting in insufficient nutritional allocation for productive growth. A transcriptomic study [26] has shown that yaks under cold-season nutritional stress would inhibit fatty acid synthesis and oxidation and promote gluconeogenesis to maintain energy balance, which is consistent with the weight-loss phenomenon of the grazing group in this study.

However, the increase in the average daily gain of yaks in the supplementary-feeding group confirmed that appropriate supplementary feeding with protein-rich concentrates (crude protein 16.24%) can effectively compensate for the deficiencies in key nutrients such as energy, protein, and minerals in natural cold-season forages. This is consistent with the conclusion proposed by Zhu et al. [27] that increasing the dietary protein level can improve the weight gain of yaks in the cold season. The latter study also found [28] that young yaks (18–30 months old) have the most significant growth response to supplementary feeding, providing a reference for the precise supplementary feeding of Yushu yaks of different ages.

This study further discovered that, relying on a balanced nutrient supply and suitable pen temperature, the pen-feeding group achieved a 16.93% weight gain, and the average daily gain of the supplementary-feeding group reached 126.91 ± 15.23 g/d. For the first time, it was clarified that different intensities of nutritional intervention have a gradient effect on the growth of Yushu yaks. This is different from the single supplementary-feeding effect reported in existing studies and provides a quantitative basis for the selection of gradient nutritional intervention strategies and feeding patterns for yaks in the cold season in practical production.

### 4.2. Effects of Different Feeding Patterns on the Blood Biochemistry of Yushu Yaks

Blood biochemical indicators can directly reflect the metabolic state and health level of an animal’s body [29]. This study, for the first time, found that the activity of total superoxide dismutase (T-SOD) in Yushu yaks shows a gradient decreasing trend of grazing group > supplementary-feeding group > pen-feeding group, and the total antioxidant capacity (T-AOC) of the supplementary-feeding group is significantly higher than that of the grazing group. This characteristic is different from the conclusion in existing studies, which only confirmed that the antioxidant enzyme activity of grazing ruminants is higher than that of pen-fed animals. A study [30] on the perinatal health of yaks found that the antioxidant capacity of yaks is closely related to the regulation of the gut microbiota, and the enrichment of short-chain fatty-acid-producing bacteria can significantly increase the SOD activity and T-AOC of yaks. This indicates that the differences in the antioxidant levels of yaks in different feeding groups in this study may also be related to changes in the gut microbiota, which is a direction worthy of further research.

Grazing yaks have long-term consumption of natural forages rich in polyphenols and continuous adaptation to the high-altitude hypoxic environment. In addition, the extreme cold stress faced by grazing and supplementary-feeding yaks during daytime outdoor activities jointly induces the enhancement of the antioxidant defense system [31,32,33]. Supplementary feeding alleviates oxidative stress through nutritional supplementation while retaining some of the yaks’ antioxidant capacity. This is not simply an extension of the conclusion that “there is a negative correlation between antioxidant enzyme activity and nutritional level”, but reveals a new regulatory feature of the antioxidant capacity of plateau-endemic yak breeds. The activity of alkaline phosphatase (ALP) in the grazing group increased significantly, which is related to the mechanical stimulation of bones from long-distance walking in the grazing mode, the metabolic regulation caused by the imbalance of the calcium–phosphorus ratio in forages, and the stimulation of intestinal ALP secretion by crude fiber [34]. This reflects the physiological adaptation of yaks to exercise and nutrient acquisition in the grazing mode. The ALP level in the pen-feeding group is higher than that in the supplementary-feeding group, which is related to the promotion of calcium and phosphorus metabolism by twice-daily feeding of high-protein concentrates, consistent with the research results of Gao et al. [35] that supplementary feeding with concentrates can significantly increase the alkaline phosphatase activity of grazing yaks.

Albumin (ALB), as a core indicator reflecting protein synthesis and nutritional status [36], showed a distribution characteristic of pen-feeding group > supplementary-feeding group > grazing group in this study. This is consistent with the research conclusion that the pen-feeding mode promotes protein synthesis and storage through a balanced nutrient supply [37,38,39]. Supplementary feeding with concentrates in the cold season can increase the serum total protein and albumin levels of grazing yaks by 12.3% and 15.6% respectively [40], which is consistent with the change trend of albumin in the supplementary-feeding group in this study. The low ALB levels in the supplementary-feeding and grazing groups are due to the nutritional limitation caused by insufficient or no concentrate supplementation. The increase in the activity of alanine aminotransferase (ALT) in the grazing group is related to the dual stresses of cold, hypoxia, and nutritional deficiency [41,42]. The relatively high ALT level in the pen-feeding group indicates that a high nutritional level combined with low exercise may increase the liver’s metabolic load. This result adds new biochemical evidence for the regulation of liver health during the cold-season feeding of Yushu yaks and is a breed-specific result not reported in existing studies on ruminant undernutrition. The higher creatinine (CREA) level in the supplementary-feeding group indicates stronger muscle creatine catabolism and more active protein metabolism, which is related to the increase in nitrogen metabolites after concentrate supplementation [43].

This study determined that Yushu yaks mainly suffer from deficiencies in sodium, phosphorus, and iron during the cold season. This mineral nutrition characteristic is different from that of ordinary grazing livestock on the Qinghai–Tibet Plateau. It is consistent with the reported conclusion that grazing livestock on the Qinghai–Tibet Plateau are prone to mineral deficiencies in the cold season, providing a specific reference for targeted mineral supplementation for this breed [44,45,46].

### 4.3. Effects of Different Feeding Patterns on the Blood Metabolism of Yushu Yaks

Blood metabolites are important indicators reflecting the nutritional and physiological status of animals and can reveal the health of body tissues and organs [47,48]. This study, for the first time, identified 2024 metabolites in Yushu yaks and found an obvious gradient effect of nutritional intervention on their metabolic phenotypes. The largest number of differential metabolites was found between the grazing group and the pen-feeding group, and the supplementary-feeding group showed an intermediate characteristic. This is a new finding not reported in previous studies on ruminant nutritional intervention, clarifying the quantitative relationship between the intensity of nutritional intervention and the metabolic phenotypes of yaks. A metabolomic study on the feeding of yaks in the warm season by Zhang et al. [49] found that the bile secretion pathway was the most significantly enriched between grazing and pen-fed yaks, which is similar to the metabolic differentiation phenomenon found in this study, indicating that the feeding pattern is a key factor leading to changes in the yak plasma metabolome.

The metabolites of the grazing group were enriched in ether lipid metabolism and the pentose phosphate pathway. Ether lipid metabolism can maintain the normal physiological function of cell membranes at low temperatures [50,51], and the pentose phosphate pathway provides nicotinamide adenine dinucleotide phosphate (NADPH) for antioxidant reactions [52]. These two, together with the elevated T-SOD level in the biochemical indicators, resist the oxidative stress caused by high-altitude low-temperature stress and jointly enhance the antioxidant capacity of grazing yaks. At the same time, the grazing group was enriched in amino-acid metabolic pathways such as glycine, serine, and threonine metabolism and fatty-acid degradation [53,54,55,56,57], indicating that when the nutrition of natural forages is unbalanced and deficient in the cold season, yaks will decompose endogenous amino acids and fats to supply energy to meet the body’s maintenance needs. This is consistent with the research results of Zheng et al. [58] that cold-season forage shortage induces hepatic fat decomposition and amino-acid decomposition in yaks. The pen-feeding group was enriched in energy-related metabolic pathways such as fatty-acid biosynthesis, lactose metabolism, and the glyoxylate cycle. Fatty-acid biosynthesis can promote fat deposition [59,60], and the glyoxylate cycle can achieve efficient energy conversion from fat to carbohydrates [61], which is consistent with the conclusion proposed by Yang et al. [62] that the standardized diet in the pen-feeding mode improves the degradation and conversion efficiency of carbohydrates.

This study, for the first time, clarified the energy-conversion synergistic pathway of the fatty-acid biosynthesis–lactose metabolism–glyoxylate cycle in Yushu yaks under pen-feeding conditions, finding that pen-fed yaks can efficiently convert fat into carbohydrates for energy to achieve rapid weight gain. This is a unique energy-metabolic characteristic of Yushu yaks different from ordinary yaks. In addition, the pen-feeding group was enriched in the cytochrome P450-mediated xenobiotic metabolism pathway [63,64], indicating an enhanced ability of pen-fed yaks to degrade xenobiotics such as feed additives. This is an important metabolic adaptation of yaks to artificial feed.

The supplementary-feeding group was enriched in the arginine synthesis, glutathione (GSH) synthesis, and primary bile acid synthesis pathways. Arginine (as a multifunctional basic amino acid) synthesis can promote ammonia excretion through the urea cycle, improve tissue oxygen supply through the nitric oxide synthase pathway, and stimulate the secretion of growth hormone to promote body growth [65,66,67]. This confirms the important role of the arginine synthesis pathway in the plateau adaptation of supplementary-fed yaks. The GSH synthesis pathway can scavenge reactive oxygen species and alleviate the oxidative damage caused by hypoxic stress [68]. The primary bile acid synthesis pathway can improve the digestion and absorption efficiency of fat [69], which is consistent with the conclusion proposed by Zhou et al. [70] that regulating lipid metabolites can improve the energy supply of yaks.

The synergistic effect of the above three pathways is the molecular mechanism by which the supplementary-feeding mode achieves a balance between the growth performance and plateau antioxidant capacity of Yushu yaks. It breaks through the existing understanding in studies that “supplementary feeding is only for nutritional supplementation” and explains the core reason why supplementary feeding takes into account the growth and plateau adaptability of yaks from the perspective of metabolic pathways.

This study has certain limitations. The experiment included an equal number of male and female Yushu yaks, yet the sex effect was not incorporated into the statistical analysis. In reality, there are differences in some growth, biochemical, and metabolic indicators between male and female individuals. Due to the scarcity of basic research data on the cold-season feeding of Yushu yaks and the fact that they are raised in mixed-sex herds locally, this study focused on the universal impact of feeding patterns on the species as a whole, thus not analyzing the sex effect. In the future, in-depth research could be conducted on the differential responses of male and female yaks to different cold-season feeding patterns, and the regulatory role of the sex factor could be analyzed to provide a theoretical basis for precise gender-specific feeding of Yushu yaks.

## 5. Conclusions

This study focused on Yushu yaks, an endemic breed living at an altitude above 4000 m, and for the first time systematically compared the multidimensional effects of three feeding modes (grazing, grazing plus supplementary feeding, and stall-feeding) on their growth performance, blood biochemistry and metabolism. In conclusion, cold-season grazing allows yaks to maintain strong antioxidant capacity and high-altitude adaptive metabolism, but causes obvious body weight loss due to insufficient nutrition. Supplementary feeding effectively improves nutritional status and growth performance while preserving antioxidant capacity. Stall-feeding significantly enhances daily weight gain but reduces antioxidant-related metabolic activity. These findings provide clear practical guidance for herders. Local producers can adopt grazing plus appropriate concentrate supplementation as the preferred strategy during the cold season. This mode balances the body weight gain, health status and adaptability of Yush yaks. For intensive breeding farms, a proper stall-feeding system can be applied to maximize growth efficiency and economic benefits. These optimized feeding patterns can effectively improve the productivity and sustainable breeding level of Yushu yaks in alpine pastoral areas.

## Figures and Tables

**Table 1:** Dietary formulations of supplementary-fed and stall-fed yaks.

Feed Composition	Maize	Wheat Bran	Soybean Meal	Rapeseed Meal	Limestone Powder	Yishengji Powder	Sodium Bicarbonate	Premix	Total
Mass Fraction (%)	40.2	35.6	13.6	4.6	0.6	0.4	1.0	4.0	100.0

Note: Per kilogram of premix provides vitamin A 300,000 IU, vitamin D3 60,000 IU, vitamin E 960 IU, vitamin B1 500 mg, nicotinamide 760 mg, copper (Cu) 500 mg, iron (Fe) 600 mg, zinc (Zn) 2250 mg, manganese (Mn) 1250 mg, cobalt (Co) 15 mg, iodine (I) 25 mg, selenium (Se) 15 mg.

**Table 2:** Nutritional levels of diets for supplementary-fed and stall-fed yaks and natural forage.

Feed Type	Comprehensive Net Energy (MJ/kg)	CP (%)	EE (%)	NDF (% DM)	ADF (% DM)	Ca (g/kg DM)	P (g/kg DM)
Diet	6.01	16.24	4.39	14.10	4.05	0.53	0.22
Natural Forage	3.54 ± 0.19	6.12 ± 0.39	2.09 ± 0.23	57.63 ± 3.40	32.53 ± 2.09	0.38 ± 0.11	0.10 ± 0.07

Note: Nutritional values of natural forages are presented as measured values ± standard deviation (SD); nutritional levels of concentrate diets are measured values. Abbreviations: CP, crude protein; EE, ether extract; NDF, neutral detergent fiber; ADF, acid detergent fiber; DM, dry matter.

**Table 3:** The effects of different feeding patterns on weight gain of Yushu yaks.

Trait	Grazing	Supplementary-Fed	Stall-Fed	CV (%)	R^2^	SEM
Initial BW/kg	277.37 ± 35.89	281.97 ± 36.41	275.47 ± 42.78	13.26	0.02	4.12
Final BW/kg	227.06 ± 30.67 b	304.81 ± 36.68 a	322.10 ± 51.59 a	16.85	0.90	6.20
ADG/(g/d)	−279.50 ± 44.45 c	126.91 ± 15.23 b	259.05 ± 61.56 a	112.58	0.99	3.87

Note: Different lowercase letters in the same row indicate significant differences among means (*p* < 0.05). SEM = standard error of the mean; CV = coefficient of variation; R^2^ = determination coefficient; Initial BW = initial body weight; Final BW = final body weight; ADG = average daily gain; *n* = 30 per group.

**Table 4:** Pairwise comparison *p* values among different feeding regimes.

Index	Grazing vs. Supplementary-Fed (*p*-Value)	Grazing vs. Stall-Fed (*p*-Value)	Supplementary-Fed vs. Stall-Fed (*p*-Value)
Initial BW (kg)	0.66	0.85	0.52
Final BW (kg)	<0.001	<0.001	0.11
ADG (g/d)	<0.001	<0.001	<0.001

## Data Availability

The data presented in this study are included in the article and/or its Supplementary Materials. For further inquiries, please contact the corresponding author directly.

## Supplementary Materials

The following supporting information can be downloaded at: https://www.mdpi.com/article/10.3390/ani16071110/s1, Table S1: Effects of different rearing modes on blood biochemical parameters of Yushu yaks; Table S2: Differential metabolites of grazing, supplementary-fed, and stall-fed groups in the positive ion mode; Table S3: Differential metabolites of grazing, supplementary-fed, and stall-fed groups in the negative ion mode; Table S4: Effects of different feeding modes on weight gain of Yushu yaks (6 yaks for biochemical detection).
