# Supplementary material for: Effects of Different Feeding Modes on Growth Performance, Blood Biochemistry, and Metabolism of Yushu Yaks During the Cold Season
Source: Animals (Basel). 2026 Apr 3;16(7):1110. doi: 10.3390/ani16071110 (PMC13072301; doi:10.3390/ani16071110)
Supplement: Supplementary file 1 [file animals-16-01110-s001.zip › animals-4170591-supplementary.pdf]

Supplementary Table S1 Effects of Different Rearing Modes on Blood Biochemical Parameters of Yushu Yaks

Item	Grazing Group	Supplementary-fed Group	Stall-fed Group
ALT（U/L）	42.33±8.74a	15.08±6.89b	45.65±13.78a
AST（U/L）	100.22±14.25a	89.32±19.07a	91.48±17.82a
CREA（μmol/L）	116.8±20.99b	168.45±50.06a	111.85±9.64b
LDH（U/L）	942.84±233.84a	1059.01±111.28a	1125.57±347.64a
TP（g/L）	65.77±2.94a	72.37±6.78a	69.77±3.59a
ALB（g/L）	38.45±1.65	41.79±3.58ab	43.76±2.05a
GLOB（g/L）	27.32±3.78a	30.58±5.85a	26.01±2.34a
GLU（mmol/L）	3.93±0.27a	5.38±1.83a	4.41±0.19a
TG（mmol/L）	0.31±0.09a	0.26±0.10a	0.27±0.06a
HDL（mmol/L）	1.76±0.45a	1.64±0.14a	1.49±0.09a
LDL（mmol/L）	0.42±0.15b	0.74±0.26a	0.74±0.07a
ALP（U/L）	24.2±11.64a	3.9±2.35b	8.23±2.75b
GGT（U/L）	8.53±2.23ab	8.02±2.05b	12.92±4.32a
Na（mmol/L）	137.56±0.98c	142.38±1.75b	144.88±1.26a
K（mmol/L）	12.95±0.45b	13.74±0.73ab	14.11±0.41a
Cl（mmol/L）	97.1±1.87a	97.9±4.62a	98.84±1.31a
P（mmol/L）	1.22±0.13b	1.76±0.35a	1.90±0.11a
Fe(umol/L)	5.04±0.11b	6.08±0.50a	5.72±0.22a
Mg（mmol/L）	0.51±0a	0.51±0a	0.51±0a
UREA（mmol/L）	4.99±0.51a	5.6±1.57a	4.72±0.77a
UA(umol/L)	46.56±8.9a	39.06±11.07a	33.66±9.31a
T-CHO（mmol/L）	2.37±0.55a	2.52±0.26a	2.33±0.11a
MDA(（μmol/L))	4.19±1.45a	3.43±0.55a	4.01±1.71a
T−SOD（U/mL)	56.74±14.7a	29.63±9.09b	11.64±3.07c
CAT（U/mL)	3.23±2.08ab	6.24±2.1a	3.01±2.16b
GSH-Px（U/mL)	10.33±3.46a	13.02±3.89a	13.72±4.53a
T-AOC（U/mL)	2.48±0.07b	2.69±0.11a	2.64±0.07a
BUN(mg/dl)	13.98±1.44a	15.68±4.39a	13.23±2.16a

Note: Values in the same row with different lowercase letters indicate significant differences ($P < 0.05$), while values with the same lowercase letter indicate no significant differences ($P > 0.05$).

Supplementary Table S2 Differential Metabolites of Grazing, Supplementary-fed, and Stall-fed Groups in the Positive Ion Mode

Compound_ID	Name	AvsB			AvsC			BvsC		
		VIP	P.value	FC	VIP	P.value	FC	VIP	P.value	FC
Com_4290_pos	Maculansin A	2.190	< 0.001	31.105	2.058	< 0.001	71.367			
Com_1314_pos	Pleuromutilin	1.958	0.011	30.820						
Com_4233_pos	Miriquidic acid	2.114	< 0.001	26.633	2.086	< 0.001	61.728			
Com_1136_pos	Ginkgolic Acid (C13:0)	1.654	0.021	24.901	1.616	0.012	49.993			
Com_1329_pos	(E/Z)-Polydatin	2.078	< 0.001	23.891	1.968	< 0.001	22.927			
Com_3500_pos	Fenethylline	2.342	< 0.001	21.464						
Com_1206_pos	Kirenol	2.041	0.005	21.028	1.854	0.004	21.329			
Com_3708_pos	Di-n-heptyl phthalate	1.952	0.015	16.783						
Com_718_pos	ascaroside C6	1.610	< 0.001	15.979	2.353	< 0.001	87.594			
Com_767_pos	Methyl linoleate	1.605	0.041	14.916						
Com_3672_pos	5-Hydroxy-3',4',7,8-tetramethoxyflavone				2.383	< 0.001	118.813	1.350	0.001	10.730
Com_3415_pos	4,5-Dihydromelampodin B				2.370	< 0.001	115.359	1.345	0.001	10.287
Com_666_pos	3-(3,4,5-Trimethoxyphenyl)propanoic acid				2.176	< 0.001	28.242			
Com_4019_pos	Gutierrezianolic acid isobutyrate methyl ester				2.238	< 0.001	18.588	1.129	0.018	3.675

Com_3784_pos	(4Z)-Tetradec-4-enoylcarnitine	2.116	0.009	26.632
Com_3919_pos	O-[(9Z)-3-hydroxytetradec-9-enoyl]carnitine	2.322	0.011	21.857
Com_1215_pos	Lactose	1.561	0.048	17.488
Com_3501_pos	6-Dodecenoylcarnitine	2.332	0.005	15.485
Com_3353_pos	4,8 Dimethylnonanoyl carnitine	1.925	0.019	14.720
Com_1137_pos	Dodecanoylcarnitine	2.330	0.003	13.597
Com_991_pos	Decanoylcarnitine	2.232	0.007	12.565

Supplementary Table S3 Differential Metabolites of Grazing, Supplementary-fed, and Stall-fed Groups in the Negative Ion Mode

Compound_ID	Name	AvsB			AvsC			BvsC		
		VIP	P.value	FC	VIP	P.value	FC	VIP	P.value	FC
Com_1279_neg	Urolithin-3-sulfate	1.652	0.008	546.479	1.184	0.018	220.927			
Com_1605_neg	Tricycloalternarene 11b	1.878	< 0.001	530.159	1.512	< 0.001	224.247			
Com_1518_neg	11-deoxy PGF2alpha	1.913	< 0.001	446.364	1.578	< 0.001	330.911			
Com_1528_neg	7alpha,8-dihydroxy-5beta,8betaH,9betaH,10alpha-Labdan-15-oic acid	1.876	< 0.001	329.672	1.536	< 0.001	278.609			
Com_1588_neg	13,14-dihydro-15-keto-PGF1alpha	1.590	< 0.001	137.199	1.678	< 0.001	387.492			
Com_242_neg	Deoxyarbutin	1.812	< 0.001	59.222	1.631	< 0.001	85.548			
Com_1974_neg	Unnarmicin A	2.047	< 0.001	40.872						
Com_488_neg	Prostaglandin E1	1.929	< 0.001	38.020	1.497	< 0.001	26.662			
Com_1213_neg	Linderagalactone B	1.620	0.006	37.264	1.446	0.005	45.622			
Com_1145_neg	moclobemide	1.650	< 0.001	35.369	1.459	< 0.001	41.855			
Com_806_neg	cis-1,2-Dihydroxy-4-methylcyclohexa-3,5-diene-1-carboxylate				1.723	< 0.001	67.444			
Com_349_neg	trans-10-Nonadecenoic acid(C19-1T)							1.893	0.002	11.071

Com_1970_neg	Tenuiphenone D	1.870	0.006	10.351
Com_1179_neg	3-hydroxy-C10-homoserine lactone	2.129	0.002	10.326
Com_411_neg	Paullinic acid	1.866	0.011	7.125
Com_305_neg	Petroselinic acid	1.939	0.127	7.004
Com_1734_neg	Spicatolide E	1.771	0.005	6.917
Com_524_neg	Phillygenin	2.071	0.003	6.870
Com_491_neg	Bavachinin	1.927	< 0.001	6.711
Com_1560_neg	12-Hydroxy-11-methoxy-8,11,13-abietatrien-20-oic acid	1.726	0.016	6.539
Com_1381_neg	Botrydial	1.321	0.048	6.411

Supplementary Table S4 Effects of Different Feeding Modes on Weight Gain of Yushu Yaks (6 Yaks for Biochemical Detection)

Trait	Grazing	Supplementary-fed	Stall-fed
Final Body Weight/kg	282..68±37.59	278.98±42.69	278.76±40.31
Average Daily Gain/(g/d)	230.29±35.83b	302.77±42.99a	324.76±52.39a
Final Body Weight/kg	-291.01±28.48c	132.18±13.60b	255.56±69.53a

Note: Different lowercase letters in the same row indicate significant differences between means ($P < 0.05$).
